# Steamed Ginger May Enhance Insulin Secretion through K_ATP_ Channel Closure in Pancreatic β-Cells Potentially by Increasing 1-Dehydro-6-Gingerdione Content

**DOI:** 10.3390/nu12020324

**Published:** 2020-01-26

**Authors:** Youn Hee Nam, Bin Na Hong, Isabel Rodriguez, Min Seon Park, Seo Yule Jeong, Yeong-Geun Lee, Ji Heon Shim, Tamanna Yasmin, Na Woo Kim, Young Tae Koo, Sang Hun Lee, Dong-Hyun Paik, Yong Joon Jeong, Hyelin Jeon, Se Chan Kang, Nam-In Baek, Tong Ho Kang

**Affiliations:** 1 Department of Oriental Medicine Biotechnology, Graduate School of Biotechnology, Kyung Hee University, Yongin 17104, Gyeonggi-do, Korea; 01030084217@hanmail.net (Y.H.N.); habina22@hanmail.net (B.N.H.); isabelula3r@gmail.com (I.R.); 01026793977@hanmail.net (M.S.P.); tjdbf26@gmail.com (S.Y.J.); lyg629@nate.com (Y.-G.L.); jee1015235@gmail.com (J.H.S.); pm.tamanna.yasmin@gmail.com (T.Y.); nawoonifty@khu.ac.kr (N.W.K.); sckang@khu.ac.kr (S.C.K.); nibaek@khu.ac.kr (N.-I.B.); 2 Kwang-Dong Pharmaceutical Co., Ltd., Seoul 06650, Korea; ytkoo@ekdp.com (Y.T.K.); spelljjt@ekdp.com (S.H.L.); 16165@ekdp.com (D.-H.P.); 3 Research Institute, Genencell Co. Ltd., Yongin 16950, Gyeonggi-do, Korea; jeyoon@genencell.co.kr (Y.J.J.); jeonhl0219@genencell.co.kr (H.J.)

**Keywords:** steamed ginger extract, diabetes mellitus, 1-dehydro-6-gingerdione, pancreatic islets, K_ATP_ channels, zebrafish, mice

## Abstract

Ginger (*Zingiber officinale* Roscoe) and its active compounds (gingerols, shogaols and paradols) have been reported as having beneficial functions for several diseases, including diabetes. In this study, we revealed that the steaming process could enhance the anti-diabetic potential of ginger. To confirm the anti-diabetic effect of steamed ginger extract (GG03), we assessed pancreatic islets impaired by alloxan in zebrafish and demonstrated anti-hyperglycemic efficacy in a mouse model. The EC_50_ values of ginger extract (GE) and GG03 showed that the efficacy of GG03 was greater than that of GE. In addition, LC_50_ values demonstrated that GG03 had lower toxicity than GE, and the comparison of the Therapeutic Index (TI) proved that GG03 is a safer functional food. Furthermore, our data showed that GG03 significantly lowered hyperglycemia in a diabetic mouse model. HPLC was performed to confirm the change in the composition of steamed ginger. Interestingly, GG03 showed a 375% increase in 1-dehydro-6-gingerdione (GD) compared with GE. GD has not yet been studied much pharmacologically. Thus, we identified the protective effects of GD in the damaged pancreatic islets of diabetic zebrafish. We further assessed whether the anti-diabetic mechanism of action of GG03 and GD involves insulin secretion. Our results suggest that GG03 and GD might stimulate insulin secretion by the closure of K_ATP_ channels in pancreatic β-cells.

## 1. Introduction

Ginger is an herbaceous perennial plant of the family Zingiberaceae that is commonly used as a spice and functional food [1]. In addition, ginger is one of the most famous medicinal herbs in traditional Medicine and is widely used to treat stomachache, arthritis, nonalcoholic fatty liver disease, primary dysmenorrhea, and nausea caused by pregnancy and chemotherapy [2,3,4,5,6]. In particular, the anti-diabetic effect of ginger and the gingerols, shogaols, and paradols is already known to improve insulin sensitivity and decrease the risk of diabetes mellitus [7,8,9,10]. The steaming process can affect chemicals and cause changes in biological activity. Steamed ginger has been shown to have a higher anti-cancer effect than fresh or dried ginger [11]. We thus hypothesized that the anti-diabetic efficacy of ginger might be enhanced by steaming.

Diabetes mellitus (DM) is a chronic metabolic disease characterized by high levels of blood glucose due to a lack of insulin secretion or insulin resistance [12,13]. Insulin is produced in pancreatic islets by β-cells [14]. In this study, we have considered whether the steaming process could influence the composition of the ginger extract and if this process could improve the therapeutic activity. Thus, we performed HPLC to confirm the change in composition of GG03. GG03 showed a 375% increase in 1-dehydro-6-gingerdione (GD) compared with GE. GD is a compound isolated from the rhizomes of *Zingiber officinale* Roscoe. The anti-diabetic effect of GD has not yet been reported. We assessed the recovery effect of GD on impaired pancreatic islets in alloxan-induced diabetic zebrafish.

Moreover, we investigated the anti-diabetic mechanisms of GG03. The most important route of insulin secretion is regulated by K_ATP_ channels and Ca^2+^ voltage-regulated channels [15,16]. The closure of K_ATP_ channels allows the secretion of insulin. Thus, we further investigated whether GG03 and GD increased insulin secretion by inhibiting K_ATP_ channels.

Finally, we further investigated GG03 activity on diabetic mice. We evaluated the effect of GG03 on hyperglycemia, pancreas mass, and diabetic biomarkers in blood serum.

In this study, we aimed to demonstrate that ginger may enhance preventive and therapeutic effects on diabetes via a steaming process in diabetic zebrafish, and to elucidate its possible mode of action. Additionally, we expected to prove the efficacy of GG03 on a diabetic mice model.

## 2. Materials and Methods 

### 2.1. Chemicals 

Alloxan, diprotin A, acarbose, suramin, α-glucosidase from *Saccharomyces cerevisiae*, p-nitrophenyl α-D-glucoside (PNPG), Gly-pro-p-nitroanilide (GPPN), Human protein tyrosine phosphatase 1B (PTP1B), para-nitrophenol phosphate (PNPP) and sea salts were purchased from Sigma-AldrichCo. (St. Louis, MO, USA). Human dipeptidyl peptidase-IV (DPP-IV) was purchased from Prospec Ltd. (Rehovot, Israel). 2-(N-(7-nitrobenz-2-oxa-1,3-diazol-4-yl)amino)-2-deoxyglucose (2-NBDG) was purchased from Thermo Fisher Scientific Inc. (Ganseville, FL, USA). Diazoxide was purchased from Santa Cruz Biotechnology Inc. (Paso Robles, CA, USA). Glimepiride was purchased from Cayman Chemical Co. Inc. (Arbor, MI, USA). 

### 2.2. Animals

Adult zebrafish were maintained with a zebrafish S type system (1500[W] × 400[D] × 2050 [H] mm) (Daejeon, Korea) and a 14-h light:10-h dark cycle at 28.5°C. Two pairs of adult zebrafish were placed in a spawning box overnight to obtain zebrafish larvae. The next day, the zebrafish spawned during a 30-min period of light. Zebrafish embryos were then collected at 3 h post-fertilization for incubation and maintained in 0.03% sea salt solution in a 14:10 h light-dark photocycle in an incubator at 28.5°C. 

Six-week-old Institute of Cancer Research (ICR) male mice were purchased from Orient Bio, Inc. (Seongnam, Korea). The mice were housed under a 12-h light:dark cycle with food and water provided ad libitum and maintained at a controlled temperature (23.0 ± 2.0°C) and humidity (50.0 ± 5.0%). 

### 2.3. Ethics Statement

All zebrafish experimental procedures were carried out in accordance with standard zebrafish protocols and were approved by the Animal Care and Use Committee of Kyung Hee University [KHUASP(SE)-15-10]. All experimental procedures using mice were carried out in accordance with protocols approved by the Animal Care and Use Committee of Kyung Hee University [KHUASP-15-17].

### 2.4. Extraction of Steamed Ginger Extract (GG03)

Fresh ginger was purchased from a local market (Suwon, Korea) and stored at room temperature. The ginger roots were rinsed with water and dried, and then 95 kg of ginger were steamed under the following conditions: 3 kgf/cm^3^, 97°C for 2 h. After drying for 40 h at 45°C in a dry oven, 11.5 kg of steamed ginger was recovered for further extraction. Steamed ginger (11.5 kg) was placed in a commercial extractor (KS220, Kyungseo Machine, Korea) with 150 L of 70% ethanol at 70°C for 5 h. The ginger extract was then filtered and concentrated. The yield was 5.65%. 

### 2.5. GE, GG03 and GD Efficacy in Treating Alloxan-Induced Pancreatic Islet Damage in Zebrafish

Zebrafish larvae (*n* = 10) were divided into the following groups: normal group, alloxan-induced group (control), and alloxan-induced groups treated with GE and GG03. Wild-type zebrafish larvae at 6 days post-fertilization (dpf) were placed into 24-well plates. The larvae were exposed to 600 μM alloxan for 3 h to induce pancreatic islet damage. To determine the efficacy of GE and GG03, the alloxan-induced larvae were treated with 1 μg/mL extracts for 3 h, then stained for 30 min with 40 μM 2-NBDG and rinsed with 0.03% sea salt solution for 20 min. After staining, pancreatic islets were observed under a fluorescence microscope (Olympus 1 × 70 microscope; Olympus Co., Tokyo, Japan) and analyzed using Focus Lite software (Focus Co, Daejeon, Korea) was used for image analysis.

### 2.6. The 50% Effective Concentration (EC_50_) of GE and GG03

Zebrafish were treated with nine different concentrations (0.01, 0.1, 0.5, 1, 5, 10, 25 and 50 μg/mL) of GE alone or GG03. The EC_50_ values were calculated by non-linear regression using GraphPad Prism version 5.01 software (Graph Pad Software, San Diego, CA, USA). 

### 2.7. The 50% Lethal Concentration (LC_50_) Values of GE and GG03

Zebrafish were treated with eight different concentrations (0.1, 1, 10, 50, 100, 125, 150 and 200 μg/mL) of either GE or GG03. LC_50_ values were calculated by non-linear regression using GraphPad Prism version 5.01 software.

### 2.8. Therapeutic Index (TI)

The TI (also referred to as the therapeutic window or safety margin) is the ratio between the toxic dose and the therapeutic dose of a drug, used as a measure of the relative safety of the drug for a particular treatment. We calculated the TI according to the following equation: TI = LC_50_/EC_50_. 

### 2.9. Quantitative Analysis of GD in GE and GG03

Calibration curves for each standard were made using six concentrations (3.125 to 100 μg/mL). GE and GG03 were filtered through 0.22 μm membrane filters (Woongki Science Co., Ltd., Seoul, Korea) and a 10 μL aliquot of each extract solution in 80% MeOH (10.0 mg/mL) was injected into the HPLC system. Formic acid was purchased from Sigma. HPLC-grade acetonitrile and water were obtained from Honeywell Burdick and Jackson Inc. (Muskegon, MI, USA). HPLC analysis was achieved using a Waters 600S (Waters, Milford, MA, USA) with a Waters 2487 UV detector (254 nm). The column was a Shimpack Gist (4.6 × 250 mm, particle size: 3 µm, Shimadzu Co., Kyoto, Japan). The mobile phase consisted of 0.1% formic acid in water (solvent A) and acetonitrile (solvent B), which were eluted at a flow rate of 0.4 mL/min with the following gradient elution with concentration of solvent 30% (0.01 min), 30% (5 min), 55% (10 min), 55% (13 min), 80% (20 min), 80% (23 min), 100% (30 min), 100% (60 min). The quantitative analysis was replicated three times.

### 2.10. Protein Tyrosine Phosphatase 1B (PTP1B) Inhibitory Activity 

A PTP1B inhibition assay was carried out according to a modified published method [17]. PTP1B activity was measured in a reaction mixture containing 10 mM para-nitrophenol phosphate (PNPP), 1 mM EDTA and 0.15 M NaCl in 100 mM sodium acetate buffer (pH 5.5). Suramin was used as positive control. One hundred microliters of reaction mixture containing PTP1B enzyme (in a concentration of 1μL/mL) were added to 100 μL of sample and then incubated for 30 min at 30 °C. The reaction was terminated by the addition of 100 μL of 100 mM NaHCO_3_. The absorbance was measured at 405 nm using a microplate reader. Experiments were done in triplicate. PTP1B inhibitory activity was expressed as inhibition (%) and was calculated as follows: Inhibition (%) = (control − sample)/control × 100.

### 2.11. Dipeptidyl Peptidase-IV (DPP-IV) Inhibitory Activity

A DPP-IV inhibition assay followed a modified published method [18]. Each sample solution was dissolved in DMSO to obtain stock solutions. Diprotin A was used as a positive control. Fifteen microliters of human DPP-IV enzyme (in a concentration of 0.05 U/mL) was added to 35 μL of the sample or vehicle in Tris-HCl buffer (50 mM, pH 7.5) in 96-well plates and then preincubated for 10 min. The enzymatic reaction was started by the addition of 50 μL of 1 mM Gly-pro-p-nitroanilide (GPPN) in Tris-HCl buffer as a substrate, and then incubated for 30 min at 37 °C. The reaction was terminated by the addition of 25 μL 25% glacial acetic acid. The absorbance was measured at 405 nm using a microplate reader. Experiments were done in triplicate. DPP-IV inhibitory activity was expressed as inhibition (%) and was calculated as follows: Inhibition (%) = (control − sample)/control × 100.

### 2.12. α-Glucosidase Inhibitory Activity

An α-glucosidase inhibition assay was carried out according to a modified published method [19]. Acarbose was used as a positive control. A volume of 60 μL of sample solution and 50 μL of 0.1 M phosphate buffer (pH 6.8) containing α-glucosidase solution (0.2 U/mL) was preincubated in 96 well plates at 37 °C for 20 min. After pre-incubation, 50 μL of 1 mM p-nitrophenyl-α-D-glucopyranoside (PNPG) solution in phosphate buffer was added to each well and incubated at 37 °C for another 15 min. The reaction was then stopped by adding 160 μL of 0.2 M NaCO_3_. Experiments were done in triplicate. Absorbance readings were recorded at 405 nm using a microplate reader. The α-glucosidase inhibitory activity was expressed as inhibition (%) and was calculated as follows: Inhibition (%) = (control − sample)/control × 100.

### 2.13. Action of diazoxide on Alloxan-Induced Diabetic Zebrafish 

Five dpf wild-type zebrafish were placed into 24-well plates. Zebrafish larvae were divided into the following twelve groups: normal, normal treated with diazoxide or alloxan, and alloxan-induced diabetic zebrafish treated with diazoxide, Glimepiride, Glimepiride + diazoxide, GE, GE + diazoxide, GE, GE + diazoxide, GG03, GG03 + diazoxide. Compounds and extracts were applied at 1 μg/mL and diazoxide at 25 μM. The zebrafish larvae were treated with 600 μM concentrations of alloxan for 3 h and then the solution was changed to 0.03% sea salt solution. After 3 h, alloxan-induced zebrafish larvae were treated (or co-treated) with respective compounds or extracts for 3 h. Following treatment, the zebrafish larvae were stained with 40 μM 2-NBDG for 30 min and rinsed with 0.03% sea salt solution for 20 min to obtain pancreatic islet images. After staining, pancreatic islet images were visualized using fluorescence microscopy and analyzed using Focus Lite.

### 2.14. Induction of DM

Following 1 week of acclimation, the mice were injected intraperitoneally with streptozotocin (STZ) (Sigma-Aldrich, St. Lois, MO, USA) at a dose of 50 mg/kg body weight to induce DM. The injection was repeated for 2 days after fasting the mice for 12 h. STZ was prepared in 0.1M sodium citrate buffer (pH 4.5) immediately before injection. The nondiabetic mice were injected intraperitoneally with saline. Mouse blood glucose levels of ≥300 mg/dL confirmed DM induction.

### 2.15. Treatment with GG03

Experimental mice were divided into four groups (*n* = 15/group). The nondiabetic ICR mice (NOR) and the STZ-induced mice (DM) were treated by oral gavage once daily with 0.5 mL of distilled water. STZ-induced mice were treated by oral gavage once daily with GG03 100 mg/kg (GG03 100) and GG03 300 mg/kg (GG03 300). Solutions containing GG03 in distilled water were prepared daily immediately prior to treatment. GG03 treatments were performed once daily for 4 weeks.

### 2.16. Blood Glucose Level Measurement

Glucose levels were determined in blood samples obtained from the tail of nonfasting mice using a strip-operated blood glucose sensor (GlucoDr.; Allmedicus Co. Ltd., Gyeonggi-do, Korea). 

### 2.17. Measurement of Biomarkers

Levels of albumin, total proteins, lactate dehydrogenase, blood urea nitrogen, creatinine, glucose, high-density lipoproteins (HDL), low-density lipoproteins (LDL), total cholesterol, and serum triglycerides in experimental groups were assessed spectrophotometrically using commercial diagnostic kits (Hoffmann-La Roche Ltd., Basel, Switzerland).

### 2.18. Statistical Analysis 

Statistical analysis was performed using Graphpad Prism (version 5). Data are expressed as mean ± standard error of mean (SEM). Differences were evaluated using repeated one-way ANOVA followed by Tukey’s test. The probability level for statistical significance was *p* < 0.05.

## 3. Results

### 3.1. Efficacy of GE and GG03 in Alloxan-Induced Diabetic Zebrafish

**Figure 1:**
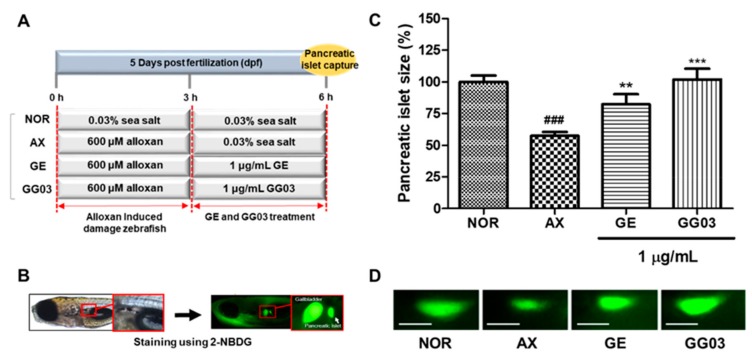
Effect of GE and GG03 on alloxan-induced pancreatic islet-damaged zebrafish. (**A**) Experimental scheme (**B**) Pancreatic islet identification using 2-NBDG dye. (**C**) Pancreatic islet size of each group. (**D**) Pancreatic islet images. (^###^ *p* < 0.001; compared to normal), (** *p* < 0.01, *** *p* < 0.001; compared to alloxan). Scale bar = 100 μm.

The average pancreatic islet size of 6 dpf zebrafish was about 2003.9 ± 339.8 μm^2^. Alloxan-induced pancreatic islet size was significantly smaller (42.5%, *p* < 0.001) than in the normal group. GE-treated pancreatic islet size was significantly increased by 24.8% (*p* = 0.002). GG03-treated groups also showed significantly increased pancreatic islet sizes (44.5%, *p* < 0.001) (Figure 1).

### 3.2. EC_50_ values of GE and GG03

**Figure 2:**
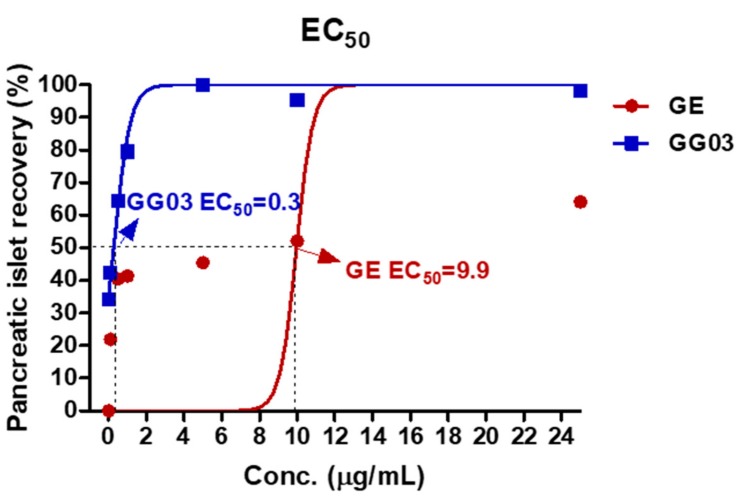
Dose-effect curves of GE and GG03. The EC_50_ of GE was 9.9 μg/mL. The EC_50_ of GG03 was 0.3 μg/mL.

We generated a dose-effect curve using zebrafish treated with GE and GG03 at eight different GE and GG03 concentrations to evaluate the EC_50_. The EC_50_ values of GE and GG03 were calculated at 9.9 and 0.3 μg/mL, respectively (Figure 2).

### 3.3. LC_50_ values of GE and GG03

**Figure 3:**
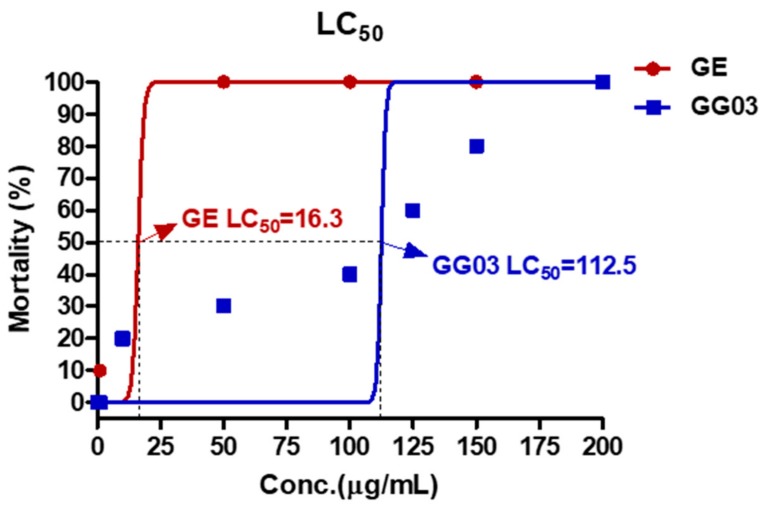
LC_50_ of zebrafish embryos exposed to GE and GG03 for 72 h. The LC_50_ of GE was 16.3 μg/mL. The LC_50_ of GG03 was 112.5 μg/mL.

We investigated mortality related to GE and GG03 in zebrafish to evaluate LC_50_. Zebrafish were treated with GE and GG03 at eight different concentrations. The LC_50_ values of GE and GG03 were calculated at 16.3 and 112.5 μg/mL, respectively (Figure 3).

### 3.4. Therapeutic Index (TI)

**Figure 4:**
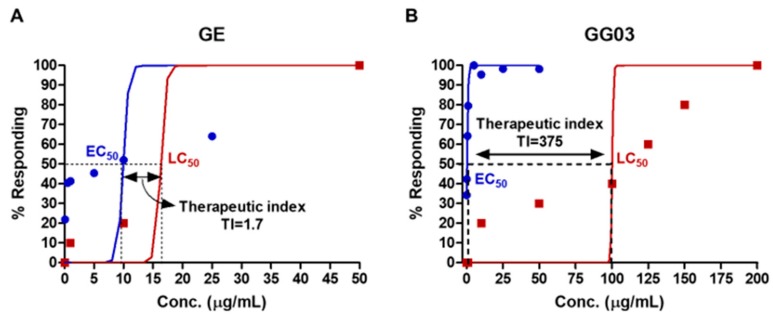
The therapeutic index (TI) of GE (**A**) and GG03 (**B**). The TI of GE and GG03 was 1.7 and 375, respectively.

The therapeutic index (TI) of GE and GG03 were calculated by LC_50_/EC_50_. The TI is used as an index of drug safety with safer drugs having a higher TI. The TIs of GE and GG03 were calculated at 1.7 and 375, respectively (Figure 4).

### 3.5. HPLC Analysis of GE and GG03

**Figure 5:**
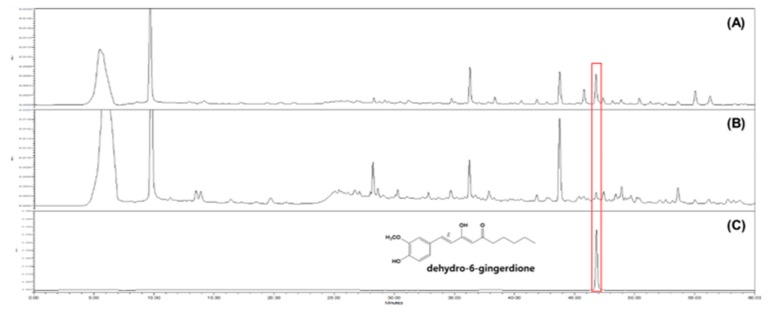
HPLC spectra of GG03 (**A**; 10,000 ppm) and GE (**B**; 10,000 ppm) and 1-dehydro-6-gingerdione (**C**; 100 ppm). HPLC analysis was carried out as described in the Materials and Methods section. Quantitative analysis was replicated three times. Chromatograms shown represent the best of three experiments.

A HPLC analysis of GE and GG03 was performed by comparing the peak area detected at 254 nm with those in calibration curves obtained using standard solutions for 1-dehydro-6-gingerdione (*y* = 3184.9*x*–6526.5, *r*^2^ = 0.9998). This compound was eluted at 46.132 min under the analysis conditions described in the Materials and Methods. The contents of 1-dehydro-6-gingerdione in the GE and GG03 were determined to be 0.04 ± 0.00% and 0.19 ± 0.01%, respectively (Figure 5). It was confirmed that the contents of GD in GG03 increased by around 375% through the HPLC experiments.

### 3.6. Efficacy of GD in Alloxan-Induced Diabetic Zebrafish

**Figure 6:**
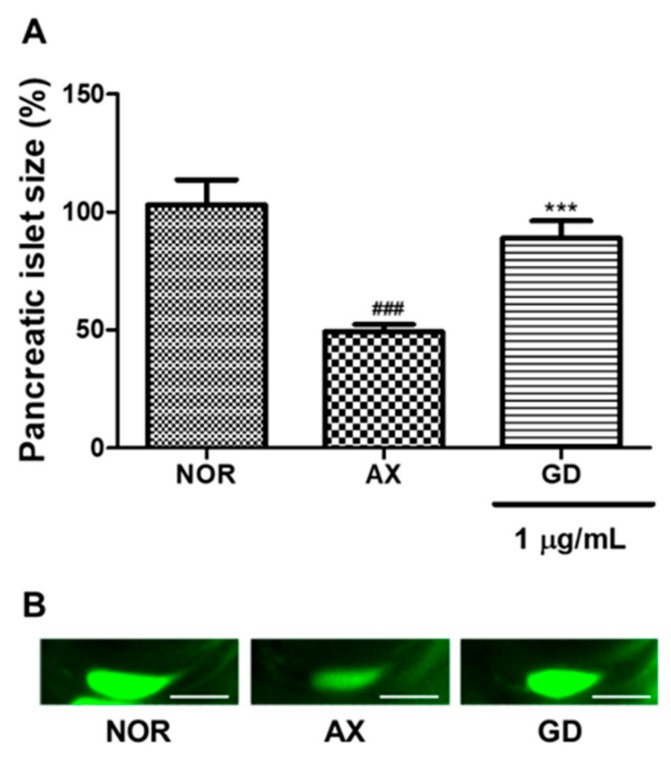
Effect of GD on alloxan-induced pancreatic islet-damaged zebrafish. (**A**) Pancreatic islet size of each group. (**B**) Pancreatic islet images. (^###^ *p* < 0.001; compared to normal), (*** *p* < 0.001; compared to alloxan). Scale bar = 100 μm.

The average pancreatic islet size of 6 dpf zebrafish was about 2023.5 ± 357.4 μm^2^. Alloxan-induced pancreatic islet size was significantly decreased (47.7%, *p* < 0.001) compared to the normal group. GD-treated groups showed significantly increased pancreatic islet size (36.1%, *p* < 0.001) (Figure 6).

### 3.7. Mechanism of Anti-Diabetes Action

The PTP1B inhibitory activities of GE, GG03 and 1-dehydro-6-gingerdione are shown in Table 1. The IC_50_ values indicated no inhibitory activity against this enzyme. The positive control for the PTP1B inhibition assay was suramin (IC_50_ = 12.3 μg/mL).

Table 2 and Table 3 show the results of DPP-IV and α-glucosidase inhibition assays, respectively, indicating that GE, GG03 and 1-dehydro-6-gingerdione showed no inhibitory activity against these enzymes. The positive control for the DPP-IV inhibition assay was diprotin A, with an IC_50_ value of 17.5 μg/mL, and the one used for the α-glucosidase inhibition assay was acarbose with IC_50_ of 1302.8 μg/mL.

### 3.8. Action on Insulin Secretion Through KATP Channels Modulation

**Figure 7:**
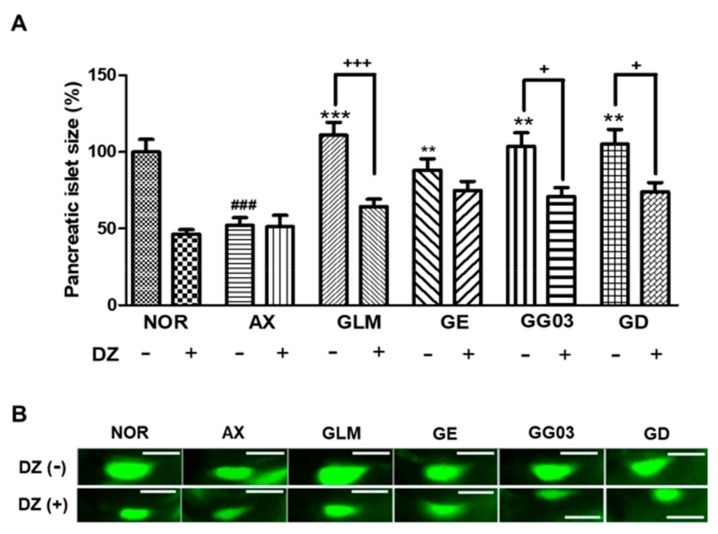
Effect of ginger extract (GE), steamed ginger extract (GG03) and 1-dehydro-6-gingerdione (GD) on K_ATP_ channels in alloxan-induced diabetic zebrafish: action of diazoxide (DZ) in the efficacy of GE, GG03 and GD. (**A**) Pancreatic islet size of each group. (**B**) Pancreatic islet images. (^###^ *p* < 0.001; compared to NOR), (* *p* < 0.05, ** *p* < 0.01, *** *p* < 0.001; compared to AX), ^++^ *p* < 0.05, ^+++^ *p* < 0.001).

Diazoxide was used as a K_ATP_ channel opener to study the involvement of pancreatic β-Cell K_ATP_ channel stimulation activity. The size of pancreatic islets in the diazoxide-treated normal group was significantly smaller (53.6%, *p* < 0.001) than in the normal group without diazoxide. Furthermore, the alloxan group showed no significant difference after treatment with 25 μM diazoxide. Pancreatic islet size was significantly decreased in the group co-treated with 10 μM glimepiride with 25 μM diazoxide (46.4%, *p* = 0.0001) compared to the group treated with 10 μM glimepiride-alone. The GE-treated group was not significantly different after treatment with 25 μM diazoxide, indicating no relationship with K_ATP_ channels. After co-treatment with GG03 and 25 μM diazoxide, the pancreatic islet size was significantly smaller (32.8%, *p* = 0.02) compared to the group treated with GG03 alone. Furthermore, the group co-treated with GD and 25 μM diazoxide had significantly decreased pancreatic islet sizes (31.2%, *p =* 0.02) compared to the group treated with GD alone (Figure 7). 

### 3.9. Body Weight and Blood Glucose Level

**Figure 8:**
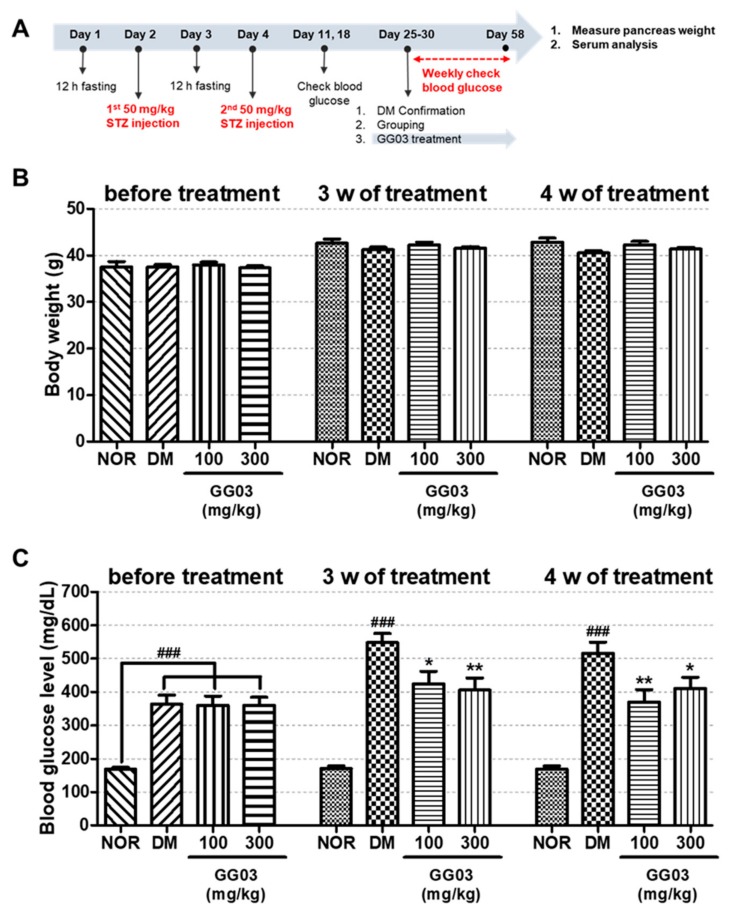
Body weights and glucose levels in diabetic mice. (**A**) Experimental scheme. (**B**) Body weight of each group. (**C**) Blood glucose level of each group. (^###^ *p* < 0.001; compared to NOR), (* *p* < 0.05, ** *p* < 0.01; compared to DM).

From 0 to 8 weeks after the STZ injection, the body weight and blood glucose levels were evaluated in all mouse groups (Figure 8). There was no significant difference in body weights. Blood glucose levels were significantly lower in the GG03 100- and 300-treated groups at 3 weeks. These results show that GG03 improved hyperglycemia in the diabetic mouse model.

### 3.10. Pancreas Weight

**Figure 9:**
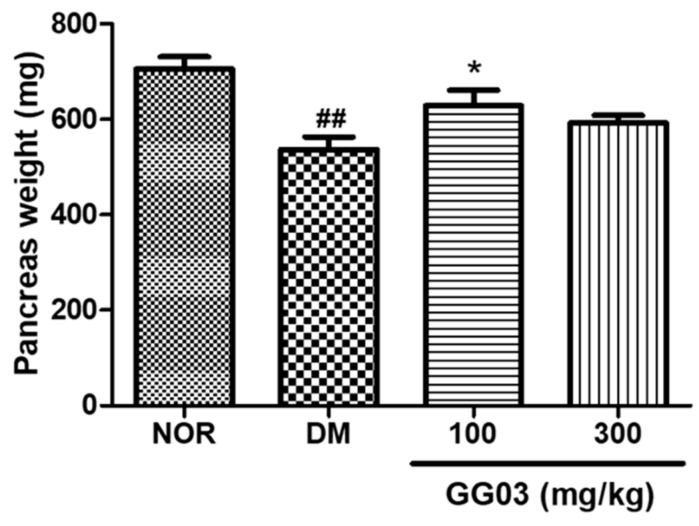
Pancreas weight at week 18 (^##^ *p* < 0.01; compared to NOR), (* *p* < 0.05; compared to DM). NOR = Normal.

The pancreatic weights of the DM group were lower than those of the NOR group, and the GG03 100-treated group had higher pancreatic weights compared to the DM group. However, pancreatic weights in the GG03 300-treated group were not significantly different to those of the DM group (Figure 9). 

### 3.11. Measurement of Biomarkers

Albumin, total protein and creatinine levels were significantly decreased in diabetic mice in comparison with normal mice. The GG03 group showed no more of a significant difference in albumin and total proteins than the DM group. Lactate dehydrogenase was increased by GG03. Creatinine and blood urea nitrogen showed no significant difference among groups (Table 4).

Glucose level was significantly higher in diabetic mice compared to normal mice. When diabetic mice were treated for 4 weeks with GG03 at 100 mg/kg, there was a significant decrease in the levels of glucose. HDL and total cholesterol in GG03 treated diabetic mice both recorded increases when compared with DM group. LDL showed no significant difference among groups. TG showed an increase when compared to normal mice, and the GG03 group reduced level of TG (Table 5).

## 4. Discussion

In this study, we evaluated whether steaming could enhance the anti-diabetic effect of GE. Generally, the heating process burns the active components, reducing the yield and physiological activity of GE. In order to overcome this disadvantage, we used a steaming process that can enhance elution of the active ingredient by indirect heating [20]. In addition, the steaming of ginger has been reported to improve its anti-cancer efficacy [11]. To confirm the enhanced anti-diabetic effect of GG03, we assessed pancreatic islets impaired by alloxan in zebrafish. To induce pancreatic islet damage in zebrafish, we used alloxan—a known diabetogenic chemical that decreases β-cell mass in pancreatic islets [21]. In a previous study, we described alloxan-induced zebrafish as a type 1 diabetes zebrafish model with decreased pancreatic islet and β-cell size [22]. We then investigated the EC_50_ of GE (EC_50_ = 9.9 μg/mL) and GG03 (EC_50_ = 0.3 μg/mL) and found that the efficacy of GG03 was greater than that of GE. We suggest that the increased amount of GD in GG03 might be responsible for enhancing the activity in pancreatic islets. In addition, LC_50_ calculations showed that GG03 (LC_50_ = 112.5 μg/mL) had lower toxicity than GE (LC_50_ = 16.3 μg/mL). Moreover, the relationship between EC_50_ and LC_50_ can be calculated as the TI, which is used in quantitative comparisons of drugs as the ratio of LC_50_ to EC_50_ [23]. The larger the TI, the safer the drug; if the TI is small, the drug must be dosed carefully and patients receiving the drug must be monitored closely for any signs of drug toxicity [23]. In this study, we demonstrated that GG03 is a safer drug relative to GE using TI values. 

HPLC was performed to confirm which component changes enhanced the efficacy of GG03. GG03 showed a 375% increase in GD compared with GE. Our data show that pancreatic islets damaged by alloxan were recovered by GD. Therefore, GG03 has been shown to enhance ginger’s beneficial anti-diabetic effects by increasing GD.

Next, to study the mechanism of GG03 and GD, we evaluated the inhibition of PTP1B, DPP-IV, and α-glucosidase, and found that GG03 had no effect any of the enzymes. PTP1B is a negative regulator of the insulin signaling pathway [24]. PTP1B inhibitors block PTP1B activity and can be used as therapy for diabetes. We expected that GG03 and GD would act as PTP1B inhibitors. However, our study demonstrates that recovery of pancreatic islet in zebrafish was not due to the PTP1B inhibitory mechanism. As one of the main mechanisms of diabetes, DPP-IV regulates postprandial glucose by the degradation of glucagon-like peptide-1(GLP-1) and Glucose-dependent insulinotropic peptide (GIP). DPP-IV can inactivate GLP-1 and GIP. Therefore, using DPP-IV inhibition increases insulin secretion, and decreases blood glucose levels [25]. GG03 and GE did not show DPP-IV inhibition. Another important pathway involved in carbohydrate metabolism is α-glucosidase enzyme, which converts disaccharides into monosaccharides at the cells lining the intestine. In diabetic patients, the short-term effect of these drugs therapies is to decrease current blood glucose levels [26]. GG03 and GD did not inhibit α-glucosidase. On the basis of these results, we investigated the pathway of insulin secretion by modulating K_ATP_ channels. Thus, we assessed whether the anti-diabetic GG03 and GD mechanism of action involves altering insulin secretion through the closure of K_ATP_ channels in pancreatic β-cells. 

The closure of K_ATP_ channels leads to membrane depolarization, the opening of voltage-gated Ca2+ channels, and Ca2+ inflow, resulting in insulin secretion [27,28]. Many diabetic models use diazoxide as an opener of K_ATP_ channels, which has been shown to inhibit insulin secretion in alloxan-induced diabetic mice and zebrafish [22,29,30]. GE has been reported to enhance the expression of glucose transporter type 4 (GLUT-4) [31]. 6-Gingerol, 6-shogaol and 6-paradolas components of ginger exhibit potent activity in stimulating glucose metabolism via the AMPKalpha2-mediated AS160-Rab5 pathway and through potentiation of insulin-mediated glucose regulation [8,32]. Furthermore, the anti-hyperglycemic action of ginger may be due to increased pancreatic secretion of insulin from β-cells [33]. Therefore, we investigated GG03- and GD-enhanced insulin secretion in diabetic zebrafish using diazoxide. Treatment with GG03 and GD in zebrafish co-treated with diazoxide was associated with significantly decreased pancreatic islet size compared to zebrafish treated with GG03 or GD alone. These results suggest that GG03 and GD might stimulate insulin secretion through closure of K_ATP_ channels in pancreatic β-cells. 

The anti-hyperglycemic effect of ginger has been previously reported in STZ-diabetic induced rats [34,35,36,37]. Thus, we evaluated the activity of GG03 on blood glucose levels in STZ-induced diabetic mice. Our results provide more evidence of the hypoglycemic efficacy of ginger extract. We showed that blood glucose levels in the GG03 groups were significantly lower after 3 and 4 weeks of treatment. After STZ-induction, blood glucose levels were significantly increased compared to normal group. Following 3 weeks of treatment, blood glucose levels in the DM group were 548 ± 100; furthermore, in the GG03 100 and GG03 300 treated mice we found similar values 424 ± 160 and 406 ± 155 respectively. Moreover, after 4 weeks of treatment, blood glucose levels in the DM group were 516 ± 115, in GG03 100 they were 369 ± 155, and in GG03 300 they were 410 ± 134; thus, GG03 100 showed greater anti-hyperglycemic effect. These results might be correlated with GG03 100, showing a protective effect on the pancreas weight of the mice. Then, since GG03 100 demonstrated greater activity, we further investigated its action on diabetic biomarkers. We did not observe any significant difference in biochemistry or lipid profiles in the serum except in glucose between diabetic mice and GG03-treated mice. 

Several references have reported that blood glucose levels in normal ICR mice are <200 mg/kg [38,39,40,41,42]. Similarly, serum levels of glucose have reported values <200 mg/kg [39]. Our serum glucose levels showed a slightly higher value than this, however, our blood glucose levels showed that normal mice were under non-diabetic ranges through all experiments. In addition to glucose values, there are other important factors that characterize diabetes disease, namely polydipsia, polyphagia and polyuria [40]. Normal mice did not present these characteristics at any time during the experiment, since in addition to checking the weekly glucose levels we also measured the amount of food and water that the mice consumed (data not shown). Therefore, slightly increased serum values in normal mice are not indicative of diabetes.

Previously, ginger has reported hypolipidemic effects [33,34], however, GG03 did not show this feature among its activity. Still, the main aim when managing diabetes type 1 remains glycemic control—to avoid complications such as diabetic neuropathies—which is mostly achieved by insulin regulation. We hypothesize that GG03 main action on glucose levels might result from the inhibitory activity on K_ATP_ channels rather than lipoproteins modulation. To focus on glycemic control, we used STZ which causes beta-cell damage by necrosis and inhibits the secretion of insulin. However, STZ is well known to induce slowly progressive diabetes in ICR mice. Previous studies have shown that 200 mg/kg of STZ injection significantly increased serum glucose levels from 1 week after STZ administration, but total cholesterol level significantly increased after 5 weeks. Moreover, 100 mg/kg of STZ injection showed a normal range of glucose level at 1 week after STZ administration which did not increase until 9 weeks after administration. Interestingly, total cholesterol level showed normal range after 9 weeks of STZ administration [43]. Perhaps our STZ induced diabetes mice are not suitable as a model to confirm hyperlipidemia improvement. Thus, further investigation of GG03 on anti-dyslipidemia mechanisms is needed.

In conclusion, this study provides compelling evidence that applying a steaming process to ginger may enhance its preventive and therapeutic effects on diabetes in diabetic zebrafish, and this enhanced activity might be related to the increased GD content. Moreover, the GG03 mode of action might result from its activity as a K_ATP_ channel inhibitor. Additionally, GG3 demonstrated anti-hyperglycemic activity on diabetic mice model.

Future studies should focus specifically on the importance of GG03 from hyperlipidemia and hyperinsulinemia as different forms of diabetes (obesity, insulin resistance).

## Figures and Tables

**Table 1:** Inhibitory effects of GE, GG03 and GD on PTP1B.

Sample	IC_50_ (μg/mL)
Suramin	12.3
GE	ND
GG03	ND
GD	ND

The half-maximal inhibitory concentration (IC_50_).

**Table 2:** Inhibitory effects of GE, GG03 and GD on DPP-IV.

Sample	IC_50_ (μg/mL)
Diprotin A	17.5
GE	ND
GG03	ND
GD	ND

The half-maximal inhibitory concentration (IC_50_).

**Table 3:** Inhibitory effects of GE, GG03 and GD on α-glucosidase.

Sample	IC_50_ (μg/mL)
Acarbose	1302.8
GE	ND
GG03	ND
GD	ND

The half-maximal inhibitory concentration (IC_50_).

**Table 4:** Biochemistry in the serum of mice.

Groups	NOR	DM	GG03
Albumin (g/dL)	3.26 ± 0.09	2.73 ± 0.35 ^++^	3.13 ± 0.5
Total proteins (g/dL)	4.90 ± 0.19	4.61 ± 0.24 ^+^	4.91 ± 0.31
Lactate dehydrogenase (U/L)	266.40 ± 118.03	270.00 ± 130.66	325.14 ± 140.75
Creatinine (g/dL)	0.21 ± 0.04	0.25 ± 0.02 ^+^	0.28 ± 0.03
Blood urea nitrogen (mg/dL)	17.78 ± 3.67	17.18 ± 2.13	14.84 ± 2.73

Data represent means ± SE (n = 7). (^+^ *p* < 0.05, ^++^ *p* < 0.01; compared to NOR). NOR: Normal; DM: Diabetes mellitus; GG03: steamed ginger extract.

**Table 5:** Lipids in the serum of mice.

Groups	NOR	DM	GG03
Glucose (mg/dL)	240.80 ± 25.84	612.50 ± 107.64 ^+++^	363.71 ± 141.09 ^***^
HDL (mg/dL)	98.00 ± 13.19	78.50 ± 26.81	92.00 ± 23.61
LDL (mg/dL)	10.40 ± 3.05	10.75 ± 6.39	13.57 ± 3.41
Total cholesterol (mg/dL)	104.60 ± 16.38	91.50 ± 29.64	103.29 ± 23.10
TG (mg/dL)	106.40 ± 41.55	207.00 ± 68.36	156.86 ± 39.69

HDL: high-density lipoproteins, LDL: low-density lipoproteins, TG: serum triglyceride. Data represent means ± SE (n = 7). (^+++^ *p* < 0.001; compared to NOR), (*** *p* < 0.001; compared to DM). NOR = Normal.

## References

[1] Attokaran M. (2017). Ginger: *Zingiber officinale* R (Zingiberaceae). Nat. Food Flavors Color..

[2] Grzanna R., Lindmark L., Frondoza C.G. (2005). Ginger—an herbal medicinal product with broad anti-inflammatory actions. J. Med. Food..

[3] Lai Y.-S., Lee W.-C., Lin Y.-E., Ho C.T., Lu K.H., Lin S.H., Panyod S., Chu Y.L., Sheen L.Y. (2016). Ginger Essential Oil Ameliorates Hepatic Injury and Lipid Accumulation in High Fat Diet-Induced Nonalcoholic Fatty Liver Disease. J. Agr. Food. Chem..

[4] Daily W., Zhang X., Kim D.S., Park S. (2015). Efficacy of ginger for alleviating the symptoms of primary dysmenorrhea: A systematic review and meta—analysis of randomized clinical trials. Pain Med..

[5] Bryer E. (2005). A literature review of the effectiveness of ginger in alleviating mild-to-moderate nausea and vomiting of pregnancy. JMWH..

[6] Panahi Y., Saadat A., Sahebkar A., Hashemian F., Taghikhani M., Abolhasani E. (2012). Effect of ginger on acute and delayed chemotherapy-induced nausea and vomiting: A pilot, randomized, open-label clinical trial. Integr. Cancer Ther..

[7] Samad M.B., Mohsin M.N.A.B., Razu B.A., Hossain M.T., Mahzabeen S., Unnoor N., Muna I.A., Akhter F., Kabir A.U., Hannan J.M.A. (2017). [6]-Gingerol, from Zingiber officinale, potentiates GLP-1 mediated glucose-stimulated insulin secretion pathway in pancreatic β-cells and increases RAB8/RAB10-regulated membrane presentation of GLUT4 transporters in skeletal muscle to improve hyperglycemia in Lepr db/db type 2 diabetic mice. BMC Complement. Altern. Med..

[8] Wei C.K., Tsai Y.H., Korinek M., Hung P.H., El-Shazly M., Cheng Y.B., Wu Y.C., Hsieh T.J., Chang F.R. (2017). 6-paradol and 6-shogaol, the pungent compounds of ginger, promote glucose utilization in adipocytes and myotubes, and 6-paradol reduces blood glucose in high-fat diet-fed mice. Int. J. Mol. Sci..

[9] Zhu J., Chen H., Song Z., Wang X., Sun Z. (2018). Effects of ginger (*Zingiber officinale Roscoe*) on type 2 diabetes mellitus and components of the metabolic syndrome: A systematic review and meta-analysis of randomized controlled trials. Evid. Based Complement. Alternat. Med..

[10] Yi J.K., Ryoo Z.Y., Ha J.J., Oh D.Y., Kim M.O., Kim S.H. (2019). Beneficial effects of 6-shogaol on hyperglycemia, islet morphology and apoptosis in some tissues of streptozotocin-induced diabetic mice. Diabetol. Metab. Syndr..

[11] Cheng X.L., Liu Q., Peng Y.B., Qi L.W., Li P. (2011). Steamed ginger (*Zingiber officinale*): Changed chemical profile and increased anticancer potential. Food Chem..

[12] Cavaghan M.K., Ehrmann D.A., Polonsky K.S. (2000). Interactions between insulin resistance and insulin secretion in the development of glucose intolerance. J. Clin. Invest..

[13] Van Belle T.L., Coppieters K.T., Von Herrath M.G. (2011). Type 1 diabetes: Etiology, immunology, and therapeutic strategies. Phys. Rev..

[14] Kim S.K., Hebrok M. (2001). Intercellular signals regulating pancreas development and function. Genes Dev..

[15] Rorsman P., Renström E. (2003). Insulin granule dynamics in pancreatic beta cells. Diabetologia.

[16] Rhodes C.J., White M.F. (2002). Molecular insights into insulin action and secretion. Eur. J. Clin. Invest..

[17] Liu F., Hill D.E., Chernoff J. (1996). Direct binding of the proline-rich region of protein tyrosine phosphatase 1B to the Src homology 3 domain of p130(Cas). J. Biol. Chem..

[18] Al-Masri I.M., Mohammad M.K., Tahaa M.O. (2009). Inhibition of dipeptidyl peptidase IV (DPP IV) is one of the mechanisms explaining the hypoglycemic effect of berberine. J. Enzyme Inhib. Med. Chem..

[19] Dewi R.T., Iskandar Y.M., Hanafi M., Kardono L.B.S., Angelina M., Dewijanti I.D., Banjarnahor S.D. (2007). Inhibitory effect of Koji Aspergillus terreus on a-glucosidase activity and postprandial hyperglycemia. Pak. J. Biol. Sci..

[20] Song C.H., Seo Y.C., Choi W.Y., Lee C.G., Kim D.U., Chung J.Y., Chung H.C., Park D.S., Ma C.J., Lee H.Y. (2012). Enhancement of antioxidative activity of Codonopsis lanceolata by stepwise steaming process. Korean J. Crop Sci..

[21] Desgraz R., Bonal C., Herrera P.L. (2011). β-Cell regeneration: The pancreatic intrinsic faculty. Trends Endocrinol. Metab..

[22] Nam Y.H., Hong B.N., Rodriguez I., Ji M.G., Kim K., Kim U.J., Kang T.H. (2015). Synergistic potentials of coffee on injured pancreatic islets and insulin action via KATP channel-blocking in zebrafish. J. Agric. Food Chem..

[23] Muller P.Y., Milton M.N. (2012). The determination and interpretation of the therapeutic index in drug development. Nat. Rev. Drug Discov..

[24] Tamrakar A.K., Maurya C.K., Rai A.K. (2014). PTP1B inhibitors for type 2 diabetes treatment: A patent review (2011–2014). Expert Opin. Ther. Pat..

[25] Thornberry N.A., Gallwitz B. (2009). Mechanism of action of inhibitors of dipeptidyl-peptidase-4 (DPP-4). Best Pract. Res. Clin. Endocrinol. Metab..

[26] Moelands S.V., Lucassen P.L., Akkermans R.P., De Grauw W.J., Van de Laar F.A. (2018). Alpha-glucosidase inhibitors for prevention or delay of type 2 diabetes mellitus and its associated complications in people at increased risk of developing type 2 diabetes mellitus. Cochrane Database Syst. Rev..

[27] Ashcroft F.M. (2007). ATP-sensitive K^+^ channels and disease: From molecule to malady. Am. J. Physiol. Endocrinol. Metab..

[28] Henquin J.C. (2000). Triggering and amplifying pathways of regulation of insulin secretion by glucose. Diabetes.

[29] Tabachnick I.I., Gulbenkian A., Seidman F. (1964). The effect of a benzothiadiazine, diazoxide, on carbohydrate metabolism. Diabetes.

[30] Nam Y.H., Le H.T., Rodriguez I., Kim E.Y., Kim K., Jeong S.Y., Woo S.H., Lee Y.R., Castañeda R., Hong J. (2017). Enhanced antidiabetic efficacy and safety of compound K⁄ β-cyclodextrin inclusion complex in zebrafish. J. Ginseng. Res..

[31] Rani M.P., Krishna M.S., Padmakumari K.P., Raghu K.G., Sundaresan A. (2012). Zingiber officinale extract exhibits antidiabetic potential via modulating glucose uptake, protein glycation and inhibiting adipocyte differentiation: An in vitro study. J. Sci. Food Agr..

[32] Lee J.O., Kim N., Lee H.J., Moon J.W., Lee S.K., Kim S.J., Kim J.K., Park S.H., Kim H.S. (2015). [6]–Gingerol Affects Glucose Metabolism by Dual Regulation via the AMPKα2–Mediated AS160–Rab5 Pathway and AMPK–Mediated Insulin Sensitizing Effects. J. Cell Biochem..

[33] Al-Amin Z.M., Thomson M., Al-Qattan K.K., Peltonen-Shalaby R., Ali M. (2006). Anti-diabetic and hypolipidaemic properties of ginger (*Zingiber officinale*) in streptozotocin-induced diabetic rats. Br. J. Nutr..

[34] Al Hroob A.M., Abukhalil M.H., Alghonmeen R.D., Mahmoud A.M. (2018). Ginger alleviates hyperglycemia-induced oxidative stress, inflammation and apoptosis and protects rats against diabetic nephropathy. Biomed Pharmacother..

[35] Abdulrazaq N.B., Cho M.M., Win N.N., Zaman R., Rahman M.T. (2012). Beneficial effects of ginger (*Zingiber officinale*) on carbohydrate metabolism in streptozotocin-induced diabetic rats. Br. J. Nutr..

[36] Ojewole J.A. (2006). Analgesic, antiinflammatory and hypoglycaemic effects of ethanol extract of *Zingiber officinale* (Roscoe) rhizomes (Zingiberaceae) in mice and rats. Phytother. Res..

[37] Bhandari U., Kanojia R., Pillai K.K. (2005). Effect of ethanolic extract of *Zingiber officinale* on dyslipidaemia in diabetic rats. J. Ethnopharmacol..

[38] Shimizu R., Sakazaki F., Okuno T., Nakamuro K., Ueno H. (2012). Difference in glucose intolerance between C57BL/6J and ICR strain mice with streptozotocin/nicotinamide-induced diabetes. Biomed. Res..

[39] Hayashi K., Kojima R., Ito M. (2006). Strain differences in the diabetogenic activity of streptozotocin in mice. Biol. Pharm. Bull..

[40] Fajardo R.J., Karim L., Calley V.I., Bouxsein M.L. (2014). A review of rodent models of type 2 diabetic skeletal fragility. J. Bone Miner. Res..

[41] Leung W., Ho F.M., Li W.P., Liang Y.C. (2017). Vitis thunbergii var. Taiwaniana leaf extract reduces blood glucose levels in mice with streptozotocin-induced diabetes. Int. J. Pharmacol..

[42] Lee S., Kim J.Y., Kim E., Seo K., Kang Y.J., Kim J.Y., Kim C.H., Song H.T., Saksida L.M., Lee J.E. (2018). Assessment of cognitive impairment in a mouse model of high-fat diet-induced metabolic stress with touchscreen-based automated battery system. Exp. Neurobiol..

[43] Ito M., Kondo Y., Nakatani A., Hayashi K., Naruse A. (2001). Characterization of low dose streptozotocin-induced progressive diabetes in mice. Environ. Toxicol. Pharmacol..

